# Feasibility and clinical outcomes of same-day discharge after robot-assisted partial nephrectomy at an ambulatory surgery center

**DOI:** 10.1007/s11701-026-03624-x

**Published:** 2026-07-20

**Authors:** Ivan Kirche-Duarte, Arie Carneiro, Salvador Jaime-Casas, Oluwatimilehin Okunowo, Anjaney Kothari, Ahmad Imam, Daniel Jiang, Rodrigo A. Fribourg-Liendo, Kelvin Zheng, Kevin G. Chan, Ali Zhumkhawala, Jonathan Yamzon, Wesley Yip, Bertram Yuh, Clayton S. Lau

**Affiliations:** 1 Department of Urology, Hospital Israelita Albert Einstein, São Paulo, Brazil; 2 Division of Urology and Urologic Oncology, Department of Surgery, City of Hope Comprehensive Cancer Center, Duarte, CA USA; 3 Department of Computational and Quantitative Medicine, Division of Biostatistics, Beckman Research Institute of City of Hope, Duarte, CA USA

**Keywords:** Same-day discharge, Robotic-assisted nephrectomy, Partial nephrectomy, Feasibility, Clinical outcomes

## Abstract

Robot-assisted partial nephrectomy (RAPN) enables the management of complex localized renal masses while preserving renal function. However, the adoption of institutionalized same-day discharge (SDD) pathways remains limited. Herein, we report the safety and feasibility of SDD RAPN at an ambulatory surgery center (ASC) at our institution. We performed a retrospective observational study of patients who underwent SDD RAPN at a freestanding ASC at our institution during 2022–2025. Patients were eligible for the SDD pathway if they had a good performance status (ECOG 0–1), localized renal masses amenable to partial resection, reliable post-discharge communication, and travel time < 2 h from their residence to the hospital. A total of 63 patients underwent 65 SDD RAPNs at our ASC during 2022–2025. Patients had a median age at surgery of 62 years (interquartile range 51–71 years), and most were male (73%), non-Hispanic (63%), White (70%), and overweight (54%). Most patients had an American Society of Anesthesiologists score of III (60%), were never-smokers (63%), and had clinical stage T1 disease (92%). Most procedures were for lesions of moderate complexity (46%). All surgical specimens revealed negative surgical margins (100%), with the most common histology being clear cell carcinoma (63%). Overall, 4/65 (6%) procedures led to any-grade Clavien-Dindo complications and 2/65 (3%) required readmissions. No deaths or local or distant recurrence were observed at a median follow-up of 1.1 years. Overall, we found that SDD is safe and feasible for appropriately selected patients undergoing RAPN, with promising perioperative outcomes and low morbidity rates.

## Introduction

Kidney cancer is one of the ten most common cancers in the US, estimated to account for more than 80,000 new cases and over 15,000 deaths in 2026 [1]. Partial nephrectomy has enabled the treatment of increasingly complex renal tumors while preserving renal function. Robot-assisted partial nephrectomy (RAPN) achieves better functional outcomes, fewer complications, lower blood transfusion rates, and a reduced risk of overtreatment compared to open partial nephrectomy [2, 3]. Despite its benefits, RAPN is still associated with potential risks of complications, such as prolonged ileus, bleeding from the surgical bed, bowel perforation, and even death within the first 72 h. For this reason, patients undergoing RAPN are generally hospitalized for one to two days postoperatively [4].

The COVID-19 pandemic introduced new challenges to perioperative care, emphasizing the need for abbreviated hospital stays to minimize infection risk and optimize resource utilization. During this period, the same-day discharge (SDD) rate after robotic urologic surgeries rose sharply in some hospitals [5]. Additionally, the increased SDD rate was not associated with higher complication or readmission rates [5, 6]. Thus, a shift toward abbreviated hospital stays for robotic procedures can be both safe and effective.

Improvements in robotic surgical techniques, combined with careful patient selection, have enabled urologic surgeons to progressively reduce length of hospital stay and explore the feasibility of SDD for patients undergoing RAPN [7]. Despite these advantages, adoption of SDD in RAPN is slow, with few centers adopting this pathway. Thus, we sought to report the clinical outcomes of outpatient SDD RAPN at an ambulatory surgery center (ASC) at our institution and demonstrate the safety and feasibility of the SDD pathway.

## Methodology

### Study design and population

We conducted a retrospective, observational study involving patients who underwent RAPN at the Outpatient Surgery Center – Amini (OSC) of the City of Hope (COH) Comprehensive Cancer Center in Duarte, California (U.S.) between January 2022 and December 2025. The OSC is a freestanding ASC on our campus, separate from the main hospital (the Helford Hospital at COH Duarte). We included patients who underwent the SDD pathway (discharged on postoperative day 0) and excluded patients who had (1) hospital stay ≥ 1 day; (2) underwent radical nephrectomy; and (3) underwent SDD RAPN at our main hospital. Patients who underwent SDD RAPN at our main hospital were excluded because of the differences in eligibility criteria for RAPN at the OSC vs. the main hospital. For example, patients undergo RAPN at our main hospital if they have a significant cardiac history, severe (class 3) obesity (body mass index or BMI ≥ 40 kg/m^2^), and/or the need for a procedure in which the single-port modality is preferred (such as retroperitoneal RAPN). All RAPNs at our OSC are performed with the multiport modality. Data were extracted retrospectively from the electronic health record. Retrospective data retrieval and analysis were approved by the COH Institutional Review Board (IRB No. 08139).

### Patient selection criteria for the SDD pathway

The SDD pathway was defined as discharge of patient on postoperative day 0. All patients undergoing the SDD pathway were required to be deemed eligible by a urologist. To maximize the safety and success of SDD following RAPN, patients were deemed eligible for this pathway only if they had (1) a good performance status (ECOG 0–1); (2) localized renal masses amenable to partial resection, (3) access to a caregiver and reliable post-discharge communication; and (4) residence within a reasonable distance from the hospital, defined as no more than a 2-hour travel time. Additionally, patients were considered ineligible for SDD if they had (1) coagulopathy or other contraindications to outpatient surgery; (2) intraoperative complications precluding early discharge; (3) a requirement for intraoperative blood transfusion; or (4) any other clinical factor deemed by the primary provider or medical team to pose a risk to SDD, including hemodynamic instability during or after the procedure.

### Surgical technique and preoperative/postoperative protocols

Multiple surgeons, all of whom were Society of Urologic Oncology (SUO)-fellowship-trained urologic oncologists, conducted the procedures included in this study. All procedures were transperitoneal in nature and were performed using the on-clamp technique. The pneumoperitoneum pressure used ranged from 12 to 15 mmHg, depending on the surgeon.

The double-layer renorrhaphy technique was used for all procedures. Collecting system entries were closed with a running suture (3 –0 V-Loc™; Medtronic plc, Minneapolis, Minnesota, USA) for the inner layer, without any selective collecting system closures or drain placement. Historically, surgeons at our institution have not used a drain in RAPNs in over a decade, although drain placement remains an option for select complex cases. The outer layer was closed with a 0 V-Loc™ (Medtronic plc) suture using the horizontal mattress technique. Hemostatic agents were selectively used during complex RAPNs with VISTASEAL™ Fibrin Sealant (Johnson & Johnson MedTech, New Brunswick, New Jersey, USA).

Both preoperative and postoperative protocols were uniform among all surgeons. Patients were prescribed no or minimal opioid pain medication during surgery or postoperatively. Intravenous ibuprofen and postoperative ibuprofen (Motrin^®^) and acetaminophen (Tylenol^®^) were prescribed instead. However, patients were given tramadol (opioid) to manage breakthrough pain as needed. Following our institutional protocols for all surgical patients, patients were given verbal and written instructions and called by the nursing staff on postoperative day 1 to conduct a wellness check. Patients were educated on the most common complications, including surgical site infection, bleeding, and pain that is refractory to analgesics, and were instructed to visit the emergency department if these symptoms appeared. Additionally, we followed our institution’s standardized postoperative template for characterizing and accounting for complications for all surgeries. Specifically, all patients were seen at 1 week, 30 days, and 90 days postoperatively to assess their clinical status and the presence of any complications.

### Data collection

Demographic and clinicopathologic variables extracted included age at surgery, sex, ethnicity, race, BMI category, American Society of Anesthesiology (ASA) score, smoking status, clinical stage, year of surgery, surgical modality, laterality, RENAL nephrometry score [8], tumor size, operative time, warm ischemia time, estimated blood loss (EBL), pathologic TNM stage, margin status, tumor histology, pain score at discharge, Clavien-Dindo complications rate [9], and readmission rate.

### Statistical analysis

Descriptive statistics were used to summarize all variables, including demographic, clinical characteristics, and perioperative outcomes. Categorical data were reported as counts and percentages. Continuous variables were reported as medians and interquartile ranges (IQRs). Follow‑up duration was estimated using the reverse Kaplan–Meier method, with time measured from the date of surgery to the date of last known follow‑up. Patients with benign tumors were excluded from follow-up analysis. All data management and statistical analyses were performed using SAS version 9.4 (SAS Institute Inc., Cary, NC, USA) and R version 4.3.0 (R Foundation for Statistical Computing).

## Results

**Fig. 1:**
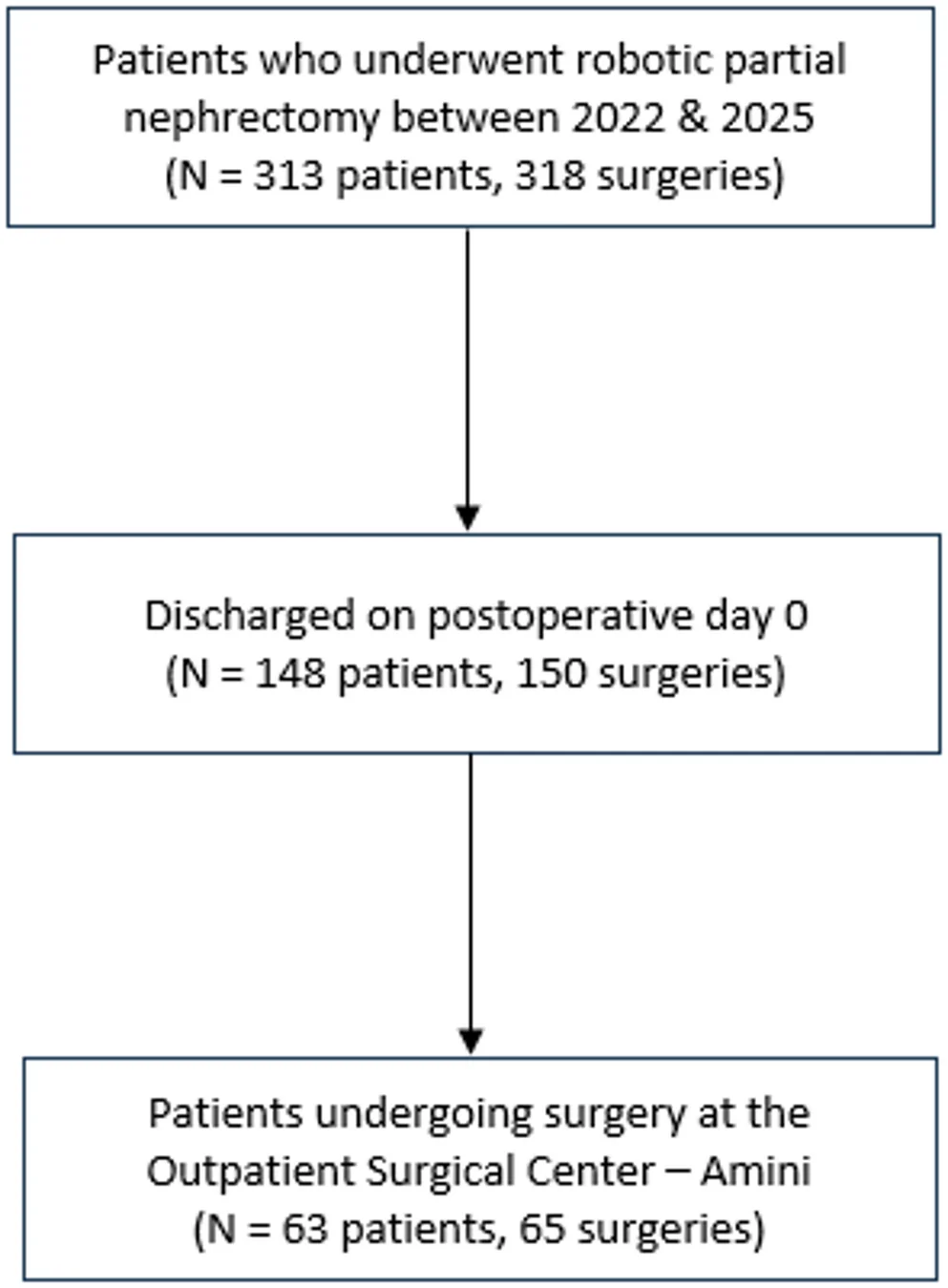
Patient selection consort diagram

Between January 2022 and December 2025, a total of 313 patients who underwent RAPN were identified (Fig. 1). Overall, 148 (47.3%) patients underwent the SDD pathway, of which 63 were treated at the OSC. Two patients underwent RAPN on two separate occasions (two surgeries each, resulting in a total of 65 eligible procedures), in which case baseline clinicodemographic data were extracted at the time of the first surgery.

Baseline clinicodemographic variables are shown in Table 1. Patients treated at the OSC had a median age at surgery of 62 years (IQR 51–71). Most patients were male (46/63; 73%), non-Hispanic (40/63; 63%), White (44/63; 70%), and overweight (34/63; 54%). Most patients had an ASA score of III (38/63; 60%), were never-smokers (40/63; 63%), and had clinical stage T1 (58/63; 92%).

**Table 1:** Baseline patient characteristics

Variable	*n* = 63^a^
**Age at surgery**,** years**	62.0 (51.0, 71.0)
**Sex**,** n (%)**	
Male	46 (73%)
Female	17 (27%)
**Ethnicity**,** n (%)**	
Hispanic or Latino	21 (33%)
Non-Hispanic or Non-Latino	40 (63%)
Unknown	2 (3%)
**Race**,** n (%)**	
American Indian or Alaska Native	1 (2%)
Asian	14 (22%)
Black or African American	3 (5%)
Native Hawaiian or Other Pacific Islander	0 (0%)
White	44 (70%)
Other	0 (0%)
Unknown	1 (2%)
**BMI category**,** n (%)**	
Normal (18.5–25.0 kg/m^2^)	14 (22%)
Overweight (25.0–30.0 kg/m^2^)	34 (54%)
Obese (≥ 30.0 kg/m^2^)	15 (24%)
**ASA**,** n (%)**	
I	4 (6%)
II	21 (33%)
III	38 (60%)
**Smoking status**,** n (%)**	
Never	40 (63%)
Current	4 (6%)
Former	19 (30%)
**Clinical stage**,** n (%)**	
T1	58 (92%)
T2	5 (8%)

^a^n (%); median (Q1, Q3)

Abbreviations: ASA, American Society of Anesthesiologists; BMI, Body Mass Index

Note: For the two patients who had more than one surgery, age and BMI were reported based on the first surgery

Surgical and perioperative outcomes are shown in Table 2. Most of the procedures were right partial nephrectomies (37/65; 57%) for lesions of moderate complexity (30/65; 46%). Surgical pathology revealed T1 (52/58; 90%), NX (36/58; 62%), and M0 (65/65; 100%) disease in most procedures (data were available only for 58/65 procedures). All surgical specimens revealed negative surgical margins (65/65; 100%), with the most common histology being clear cell carcinoma (41/65; 63%), followed by chromophobe (7/65; 11%), and papillary (5/65; 8%). The median pain score at discharge was 2 (IQR 0–5).

**Table 2:** Surgical, perioperative, and postoperative characteristics

Variable	*n* = 65^a^
**Surgery year**,** n (%)**	
2022	2 (3%)
2023	20 (31%)
2024	22 (34%)
2025	21 (32%)
**Laterality**,** n (%)**	
Left	28 (43%)
Right	37 (57%)
**RENAL nephrometry score**,** n (%)**	
Low Complexity	22 (34%)
Moderate Complexity	30 (46%)
High Complexity	13 (20%)
**Tumor size**,** cm**	2.9 (2.2, 4.2)
**Operative time**,** minutes**	166.0 (133.0, 197.0)
**Warm ischemia time**,** minutes**	22.0 (17.0, 26.5)
Missing	5
**EBL**,** ml**	50.0 (50.0, 100.0)
**Pathologic T stage**,** n (%)**	
T1	52 (90%)
T2	2 (3%)
T3	4 (7%)
Missing	7
**Pathologic N stage**,** n (%)**	
N0	22 (38%)
NX	36 (62%)
Missing	7
**Pathologic M stage**,** n (%)**	
M0	65 (100%)
**Surgical margin status**,** n (%)**	
Negative	65 (100%)
Positive	0 (0%)
Unknown	0 (0%)
Missing	0
**Histology**,** n (%)**	
Clear cell	41 (63%)
Papillary	5 (8%)
Chromophobe	7 (11%)
Mixed	1 (2%)
Angiomyolipoma	2 (3%)
Oncocytoma	5 (8%)
Other	4 (6%)
Benign/No tumor	0 (0%)
**Pain score at discharge**	2 (0, 5)
**Clavien-Dindo complication**,** n (%)**	4 (6%)
**Readmission**,** n (%)**	2 (3%)

^a^n (%); median (Q1, Q3)

N = 65 procedures because of patients who had multiple surgeries

Abbreviations: EBL, estimated blood loss

Overall, 4/65 (6%) of the procedures resulted in any-grade Clavien-Dindo complications, and readmissions occurred in 2/65 (3%) procedures. Two patients experienced grade ≥ 3 Clavien-Dindo complications, including gastrointestinal and vascular complications (Table 3). Both patients required readmission to the hospital. Median follow-up was 1.1 years (95% confidence interval or CI 0.9–1.6), with no patient experiencing death, or local or distant recurrence.

**Table 3:** Grade ≥ 3 Clavien-Dindo complications

Age at Surgery	Days from Surgery	Complication	Complication Grade	Complication Category	Readmitted
72	322	Incisional Hernia	3	Gastrointestinal	Yes
63	14	Pseudoaneurysm	3	Hemorrhagic/vascular	Yes

## Discussion

Through this retrospective study, we aimed to describe our institutional experience with the SDD pathway at an outpatient surgery center for patients undergoing RAPN, focusing on the safety and feasibility of this pathway in a freestanding ASC. We observed low rates of complications and readmission after the SDD pathway, and no mortality or recurrence in our cohort during the follow-up period. Adding to the growing body of literature in this area, our institutional experience suggests that SDD after RAPN is safe and feasible in appropriately selected patients.

There has been a gradual shift in the field toward outpatient RAPN, as it is increasingly being considered a safe and feasible pathway for appropriately selected patients, especially when performed by experienced surgeons who can achieve reliably low rates of readmissions and major complications [10]. Some recent prospective real-world studies have even demonstrated a successful planned SDD rate of up to 97% of patients undergoing RAPN, with the success rate reaching 100% by the third year of implementation [6, 7]. Additionally, SDD after RAPN is associated with significant economic advantages [11, 12] and high patient satisfaction [13, 14]. However, a systematic review and meta-analysis evaluating the safety and feasibility of ambulatory minimally invasive partial nephrectomy (MIPN) (*N* = 11 studies) found that only 1,419 (7%) of the pooled total population of 20,548 patients had a length of stay less than one day [15]. Thus, the overall proportion of patients receiving SDD remains quite small.

The percentage of patients who were deemed eligible for the SDD pathway in our study is greater than or comparable to some previous reports, such as those by Wald et al. (14.4%) [12], Mehrazin et al. (27.4%) [10], and Wood et al. (38.5%) [11]. The cases in our study were well distributed across low, moderate, and high complexities, demonstrating that the implementation of the SDD pathway was feasible irrespective of tumor complexity. In a similar study, Abaza and Shah found discharge on postoperative day 1 after RAPN to be feasible irrespective of tumor complexity [16].

Notably, all patients in our outpatient SDD cohort underwent RAPN using the multiport robot-assisted technique. This was because the OSC at our institution is equipped only with the multiport robot, while both single-port and multiport modalities are available for patients treated at our main hospital. However, the single-port RAPN is associated with a shorter length of stay and may thus be more amenable to the SDD pathway than the multiport procedure [17]. Others have found the SDD rate to be similar between patients undergoing single-port and multiport RAPN [18]. In the future, we plan to conduct a retrospective comparative analysis of the perioperative outcomes of patients undergoing SDD after single-port vs. multiport RAPN at our main hospital.

Hemorrhagic complications are among the most common complications associated with RAPN, but their overall incidence remains low (< 6%) [19–21]. When bleeding does occur after RAPN, it could be intraoperative, early postoperative (within hours of surgery), or delayed postoperative (1–2 weeks postoperatively) [19, 22]. At our institution, any patient with signs of hemodynamic instability intraoperatively or in the early postoperative period is admitted and deemed ineligible for the SDD pathway. Only one case of delayed postoperative hemorrhagic complication (pseudoaneurysm) was noted in our cohort, resulting in readmission two weeks postoperatively. It is important to note that severe hemorrhagic complications may be life-threatening [21], making it critical that the duration of admission and observation after RAPN be determined by urologists on a case-to-case basis.

Overall, the complication and readmission rates observed in our cohort were low and comparable to those noted in previous reports. For example, Wald et al. reported a 4.8% Clavien-Dindo complication rate and a 4.8% readmission rate among 21 patients undergoing SDD after RAPN [12]. Meanwhile, Benamran et al. observed a 10% Clavien-Dindo complication rate and a 10% 30-day readmission rate in 20 patients undergoing SDD after RAPN [14]. However, these results should be considered in light of the limitation of heterogeneity in the follow-up period across patients in our cohort (and the overall short median duration of follow-up), which may have masked the true complication and readmission rates as well as incidence of mortality and recurrence in our study. Longer follow-up periods are needed in the future to further establish the feasibility and relevance of SDD in outpatient RAPN. Future studies should also focus on the patient experience to determine postoperative satisfaction rates among patients undergoing SDD RAPN.

## Conclusions

SDD after RAPN was safe and feasible at our institution’s outpatient center. Over a three-year initial period, we noted promising perioperative outcomes, including low complication and readmission rates and no recurrence or mortality during the follow-up period, despite operating on tumors of low, moderate, as well as high complexities. Future research, including prospective studies, evaluating the widespread adoption of SDD pathways is necessary to validate these results.

## Data Availability

All data supporting the findings of this study are available within the paper.
