# Correction: Feasibility and clinical outcomes of same-day discharge after robot-assisted partial nephrectomy at an ambulatory surgery center

**DOI:** 10.1007/s11701-026-03999-x

**Published:** 2026-10-05

**Authors:** Ivan Kirche-Duarte, Arie Carneiro, Salvador Jaime-Casas, Oluwatimilehin Okunowo, Anjaney Kothari, Ahmad Imam, Daniel Jiang, Rodrigo A. Fribourg-Liendo, Kelvin Zheng, Kevin G. Chan, Ali Zhumkhawala, Jonathan Yamzon, Wesley Yip, Bertram Yuh, Clayton S. Lau

**Affiliations:** 1 Department of Urology, Hospital Israelita Albert Einstein, São Paulo, Brazil; 2 Division of Urology and Urologic Oncology, Department of Surgery, City of Hope Comprehensive Cancer Center, Duarte, CA USA; 3 Department of Computational and Quantitative Medicine, Division of Biostatistics, Beckman Research Institute of City of Hope, Duarte, CA USA

**Correction: Journal of Robotic Surgery**

**https://doi.org/10.1007/s11701-026-03624-x**

The original version of the article carried the following statement under the subheading “Surgical technique and preoperative/postoperative protocols” of the Methods section:

“Multiple surgeons, all of whom were Society of Urologic Oncology (SUO)-fellowship-trained urologic oncologists, conducted the procedures included in this study.”

However, this statement should have been:

“Multiple surgeons, all of whom were fellowship-trained urologic oncologists, conducted the procedures included in this study.”

